# Clinical applications of circulating biomarkers in non-small cell lung cancer

**DOI:** 10.3389/fcell.2024.1449232

**Published:** 2024-08-21

**Authors:** Hyun-Ji Oh, Abdulhamid B. Imam-Aliagan, Yeo-Bin Kim, Hyun-Jin Kim, Issac A. Izaguirre, Chang K. Sung, Hyungshin Yim

**Affiliations:** ^1^ Department of Pharmacy, College of Pharmacy, Hanyang University, Ansan, Gyeonggi-do, Republic of Korea; ^2^ Department of Biological and Health Sciences, College of Arts and Sciences, Texas A&M University-Kingsville, Kingsville, TX, United States

**Keywords:** non-small cell lung cancer, circulating factors, microRNA, exosome, ctDNA, diagnostic tools, cancer stem cells

## Abstract

Despite recent advances in cancer diagnostics and treatment, the mortality associated with lung cancer is still the highest in the world. Late-stage diagnosis, often accompanied by metastasis, is a major contributor to the high mortality rates, emphasizing the urgent need for reliable and readily accessible diagnostic tools that can detect biomarkers unique to lung cancer. Circulating factors, such as circulating tumor DNA and extracellular vesicles, from liquid biopsy have been recognized as diagnostic or prognostic markers in lung cancer. Numerous clinical studies are currently underway to investigate the potential of circulating tumor DNA, circulating tumor RNA, exosomes, and exosomal microRNA within the context of lung cancer. Those clinical studies aim to address the poor diagnostics and limited treatment options for lung cancer, with the ultimate goal of developing clinical markers and personalized therapies. In this review, we discuss the roles of each circulating factor, its current research status, and ongoing clinical studies of circulating factors in non-small cell lung cancer. Additionally, we discuss the circulating factors specifically found in lung cancer stem cells and examine approved diagnostic assays designed to detect circulating biomarkers in lung cancer patients.

## 1 Introduction

**FIGURE 1:**
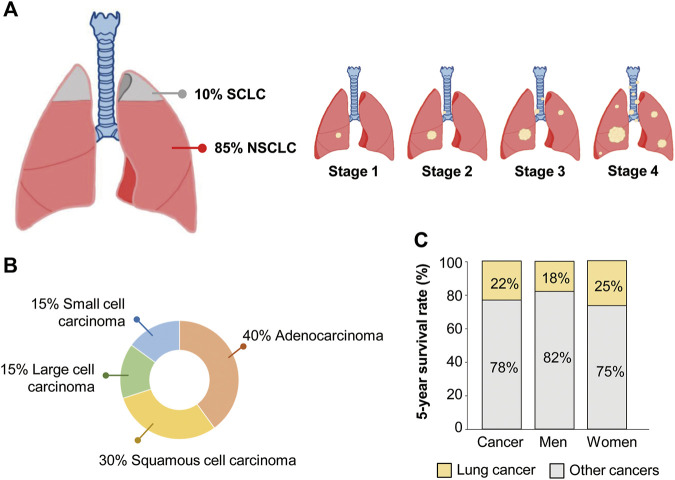
Clinical statistics of non-small cell lung cancer (NSCLC) patients. **(A)** Proportion of NSCLC and small cell lung cancer (SCLC) among total lung cancer cases. **(B)** Proportion of three different types of NSCLC and SCLC. **(C)** The 5-year survival rate of lung cancer patients.

Non-small cell lung cancer (NSCLC) accounts for 85% of all lung cancers (Zappa and Mousa, 2016). Lung cancer is classified into small cell lung cancer (SCLC) and NSCLC according to histopathologic criteria, such as the size and shape of cancer cells (Zappa and Mousa, 2016) (Figure 1A). Non-small cell cancer can be divided into three types, with adenocarcinoma accounting for 40%, squamous cell carcinoma accounting for 30%, and large cell carcinoma accounting for 15% (Figure 1B). Lung adenocarcinoma and squamous cell carcinoma occur frequently in the lungs and bronchi, respectively (Zappa and Mousa, 2016). Lung cancer is the most frequent cause of cancer death in the United States (Siegel et al., 2022). The 5-year survival rate for all people with any type of lung cancer is approximately 22%, 18% for men and 25% for women (Figure 1C). Lung cancer has the highest mortality rate among cancers because about half of the patients are diagnosed in the fourth stage, when metastases to other tissues have already progressed. Between 55% and 80% of NSCLC patients have locally advanced or metastasized disease at their initial diagnosis (Sung et al., 2021; Steeg, 2016). In addition, it has a high rate of recurrence or metastasis and a low cure rate (Steeg, 2016). Chemotherapy and radiation therapy are the main treatment modalities because NSCLC can be treated surgically in the early stage of cancer. However, detecting the early stage of lung cancer is difficult. Even when surgery is an option, the recurrence rate after surgery is high, 20%–45% (Chen et al., 2021). The high rates of lung cancer recurrence and therapy resistance are due, in part, to cancer stem cells (CSCs), which have the characteristics of self-renewal, multidirectional differentiation, and unlimited proliferation (Rowbotham et al., 2022). Because CSCs can lead to chemotherapy resistance, strong tumorigenicity, invasion, and metastasis (Rowbotham et al., 2022), diagnostic assays based on CSC-specific factors would have a significant clinical impact. The limitations of lung cancer treatment and poor diagnostics indicate a need to develop better diagnostic markers. Circulating factors detectable in a liquid biopsy could be suitable diagnostic or prognostic markers of lung cancer.

Although traditional tissue biopsies are essential for diagnosing and characterizing cancer types, they carry a significant risk of complications such as infection and damage to surrounding normal tissues, and those risks are elevated for patients with pre-existing health conditions. Moreover, when tumors are located in challenging areas of the body, an invasive biopsy might not be a feasible option. Therefore, cancer biomarkers circulating in the blood are being investigated to increase the sensitivity of diagnostics and prognostics for cancer. Circulating biomarkers can be from cancer-derived exosomes; circulating tumor RNA (ctRNA) such as microRNA (miRNA) and non-coding RNA; circulating tumor cells (CTCs); circulating tumor DNA (ctDNA); and cell-free DNA (cfDNA). In this review, we discuss the characteristics of each of those factors along with its current research status and current clinical studies in NSCLC. Additionally, we discuss the circulating factors specifically found in lung CSCs and explore approved diagnostic assays that are already being used to detect circulating biomarkers of lung cancer.

## 2 Circulating factors

**FIGURE 2:**
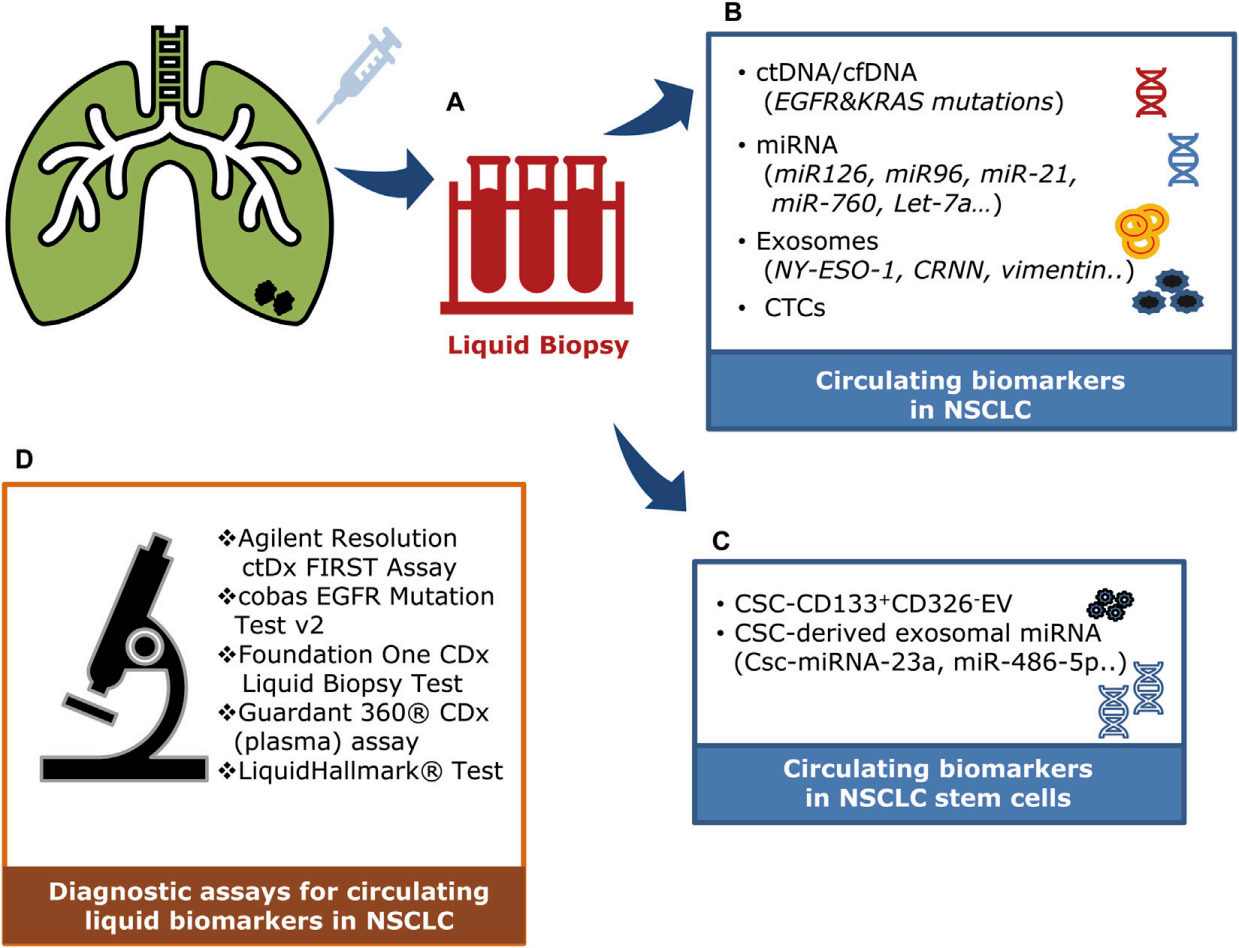
Clinical applications of circulating factors in NSCLC. **(A)** A liquid biopsy method using blood samples (plasma/serum) to detect and monitor circulating factors in NSCLC. **(B)** Circulating tumor DNAs (ctDNA/cfDNA), microRNAs, exosomes, and circulating tumor cells can be detected in the plasma of NSCLC patients. **(C)** Circulating stem cell–derived exosomal microRNAs and extracellular vesicles (CD133^+^CD326^-^). **(D)** Diagnostic assays are being used to detect circulating liquid biomarkers in NSCLC patients.

Cancer prognosis, monitoring, and treatment have always been based on tumor biopsies assessed at the time of initial diagnosis. However, that invasive and limited approach cannot be used to monitor ever-evolving tumor dynamics. Recent developments in treatments for NSCLC have led to the use of non-invasive diagnostic methods such as liquid biopsies (Lim et al., 2018). The liquid biopsy method is a minimally invasive approach to cancer diagnosis that analyzes circulating molecules such as ctDNA, cfDNA, circulating tumor miRNA, and extracellular vesicles (exosomes) (Table 1; Figure 2) (Lim et al., 2018; Bettegowda et al., 2014; Ou et al., 2018). These circulating markers are readily accessible in body fluids such as plasma, serum, cerebrospinal fluid, saliva, and urine (Bettegowda et al., 2014). Due to its minimal invasiveness, liquid biopsy is emerging as an important model for detecting circulating biomarkers associated with various forms of cancer, and it is continuously being developed as a reliable model for understanding the dynamics of tumor progression and exploring possible treatment approaches (Ou et al., 2018).

**TABLE 1:** Circulating biomarkers in NSCLC patients.

Biomarker	Types	Methods	Stage	Reference
EGFR mutation	ctDNA	Deep sequencing	Early	Mok et al. (2015), Zhang et al. (2018)
KRAS mutation	ctDNA	Digital droplet PCR	Early	Guibert et al. (2016)
Methylated *SHOX2, RARB2*	ctDNA	Quantitative methylation-specific PCR	Advanced	Kneip et al. (2011), Ponomaryova et al. (2013)
NY-ESO-1	Exosome	Proteomic analysis	—	Sun et al. (2018)
CRNN	Exosome	Proteomic analysis	—	Sun et al. (2018)
BPIFA1	Exosome	Proteomic analysis	—	Sun et al. (2018)
MUC5B	Exosome	Proteomic analysis	—	Sun et al. (2018)
IQGAP	Exosome	Proteomic analysis	—	Sun et al. (2018)
vimentin	Exosome	Immunostaining	Advanced	Rahman et al. (2016)
mi-R126	microRNA	qRT-PCR	Advanced	Grimolizzi et al. (2017)
mi-R96	microRNA	qRT-PCR	Advanced Cisplatin -resistance	Wu et al. (2017)
miR-200c-3p	microRNA	NGS, qRT-PCR	Advanced	Geng et al. (2023)
miR-4429	microRNA	NGS, qRT-PCR	Advanced	Geng et al. (2023)
miR-601	microRNA	qRT-PCR	Early	El-Aal et al. (2023)
miR-760	microRNA	qRT-PCR	Early	El-Aal et al. (2023)
miR-222-3p	microRNA	Microarray, qRT-PCR	Gemcitabine-resistance	Wei et al. (2017)
miR-21	microRNA	Microarray, qRT-PCR	Advanced	Pop-Bica et al. (2020), Dejima et al. (2017)
Let-7a	microRNA	qRT-PCR, nano testing	Advanced	Pop-Bica et al. (2020)
miR-1246	microRNA	qRT-PCR	Stage I-III	Zhang et al. (2016)
miR-1290	microRNA	qRT-PCR	Stage I-III	Zhang et al. (2016)
miR-21-5p	microRNA	qRT-PCR	Early	Tang et al. (2021)
miR-126-3p	microRNA	qRT-PCR	Early	Di Paolo et al. (2021)
miR-221-3p	microRNA	qRT-PCR	Early	Di Paolo et al. (2021)
miR-155-5p	microRNA	qRT-PCR	Early	Zhu et al. (2020)
miR-223-3p	microRNA	Blotting analysis	Early	Zhu et al. (2022)
miR-200b/c	Exos-miRNA	qRT-PCR	Advanced	Tang et al. (2018)
miR-660-5p	Exos-miRNA	qRT-PCR	—	Yongjian Qi and Zhang (2019)
miR-23a	Exos-miRNA	qRT-PCR	—	Han et al. (2017), Hsu et al. (2017), Aramini et al. (2020)
miR-128	microRNA	qRT-PCR	—	Jiang et al. (2016), Hu et al. (2014), Pan et al. (2018)
miR-4257	microRNA	qRT-PCR	Advanced	Dejima et al. (2017)
miR-486-5p	microRNA	qRT-PCR	Early	Moro et al. (2022)

### 2.1 Circulating tumor DNA and cell-free DNA

**FIGURE 3:**
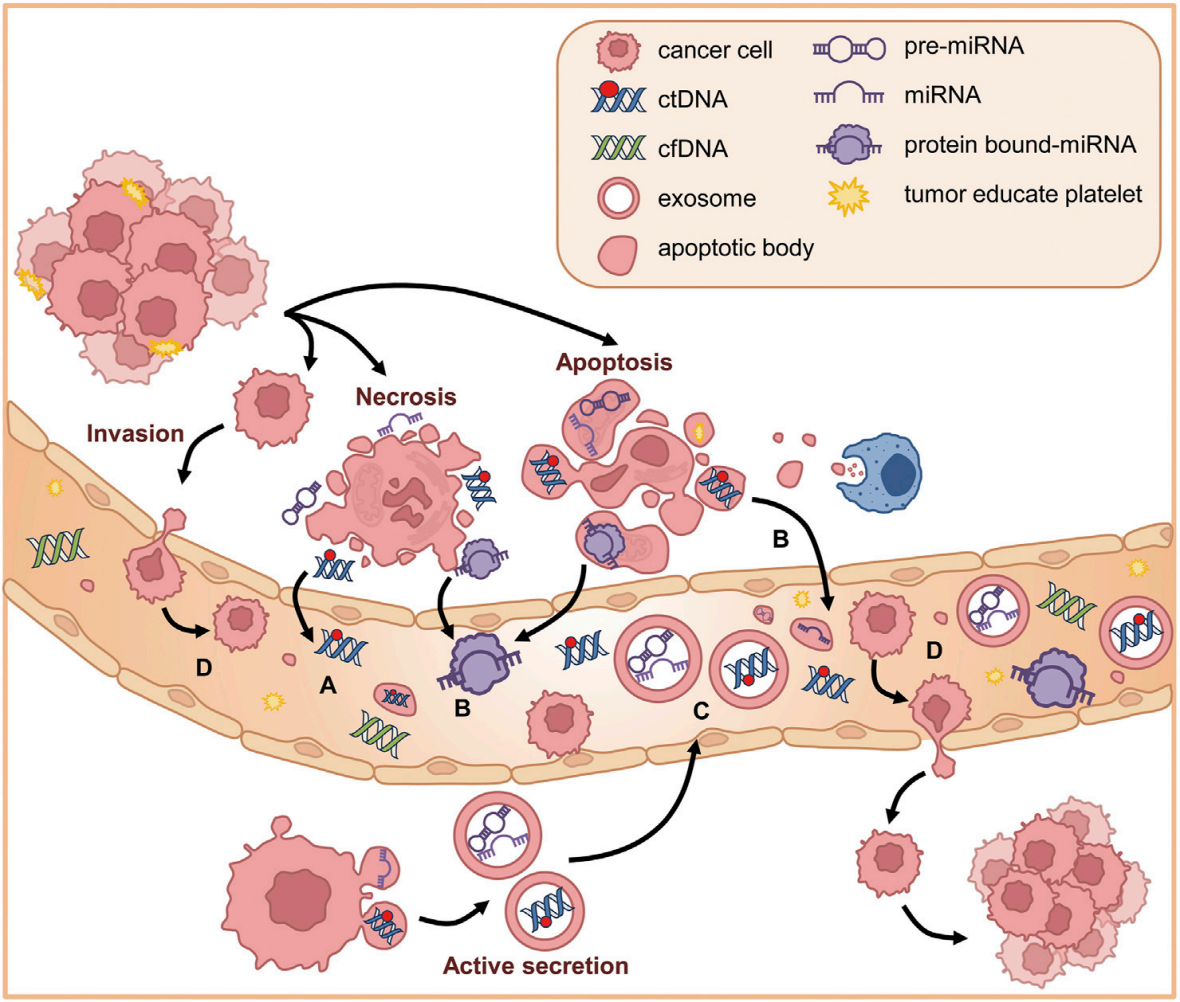
Circulating factors in NSCLC. **(A)** ctDNAs are released into the bloodstream through several physiological processes including necrosis, apoptosis, and active secretion. **(B)** In plasma, the extracellular RNAs (mRNA, miRNA, long non-coding RNA, and other types of RNA) are secreted into plasma through protein–mRNA complexes, exosomes, tumor-educated platelets, or apoptotic bodies. **(C)** Exosomes are small vesicles released through exocytosis and can be secreted into body fluids. Exosomes derived from tumor cells can transport oncogenic ctDNA and miRNA. **(D)** CTCs are tumor cells from metastatic cancer cells that detach from the primary site, invade the neighboring extracellular matrix, enter the bloodstream, and circulate in the vascular system until they extravasate and form secondary tumors.

cfDNA is a mixture of double-stranded nucleic acid fragments 150–200 base pairs in length. ctDNA is one of cfDNA derived from tumor cells having mutant DNA. They are released into the bloodstream through several physiological processes: apoptosis, cell division, necrosis, and active secretion (Figure 3A) (Liao et al., 2011; Mouliere et al., 2018). The presence of cfDNA was first described in 1948 by Mandel and Metais (Mandel and Metais, 1948). The normal average level of cfDNA is low in healthy individuals (10–15 ng/mL). cfDNA is released into the bloodstream from various sources, including endothelial tissues and peripheral blood mononuclear cells, and is typically cleared within 1 h of release (Catarino et al., 2012). However, the level is significantly elevated in patients with lung cancer as a result of tissue stress conditions such as surgery, inflammation, tissue damage, and necrosis, which are typical hallmarks of cancer (Catarino et al., 2012). The circulating mutant DNA that is released by apoptotic and necrotic cells is called ctDNA, and it carries genetic information about the tumor genome. The amount of ctDNA released into the bloodstream, which can vary from 0.01% to 90% of total cfDNA, depends on various factors, including the tumor’s location, size, degree of metastasis and progression, vascular extravasation, and stage (Diehl et al., 2008). When circulating in the bloodstream, ctDNA has a half-life ranging from 15 min to 2 h. This short window enables ctDNA to be an effective real-time model of the tumor and a possible predictor of the prognosis of NSCLC at different stages (Diehl et al., 2008).

During an evaluation of plasma as a potential diagnostic tool for early-stage lung cancer, researchers found that patients with NSCLC had high levels of ctDNA (Bettegowda et al., 2014). Newman et al. revealed 100% detection of elevated ctDNA in patients with stage II–IV NSCLC and detection in approximately half of those with NSCLC at an earlier stage (Newman et al., 2014). Several studies have shown that high concentrations of cfDNA/ctDNA have a significant link to the overall survival (OS) times of patients with NSCLC. Elevated cfDNA plays a vital role in identifying the blood mutational burden in NSCLC, and analyzing mutations could enable the identification of specific mutations and molecular targets for therapies (Ou et al., 2018). For example, next-generation sequencing (NGS) of plasma ctDNA can be used to detect *ALK* gene rearrangements and fusion genes (Lin et al., 2021). ALK is a membrane-bound tyrosine kinase belonging to the insulin receptor superfamily. Rearrangements of *ALK* have been observed in almost 10% of NSCLC patients (Rosas et al., 2019), prompting researchers to conduct studies using ALK tyrosine kinase inhibitors as a novel treatment for lung cancer (Cicin et al., 2022). In addition to *ALK* rearrangement, ctDNA is also linked to epidermal growth factor receptor (EGFR) mutations. Advanced lung cancer patients with EGFR mutations typically show hypersensitivity to the tyrosine kinase inhibitors (TKIs) gefitinib and erlotinib (Lynch et al., 2004; Paez et al., 2004; Rosell et al., 2009). Therefore, detecting EGFR mutations from liquid biopsy samples enables personalized and targeted treatment. A study investigating ctDNA as a substitute for invasive tumor biopsy linked *EGFR* mutation in ctDNA to cancer prognosis (Buder et al., 2021). An analysis of patients with an EGFR mutation showed that the presence of an L858R mutation in tumor tissue was an indicator of poor OS times (Buder et al., 2021). Genome-wide assays and sequencing have been conducted to identify possible alterations. Single-nucleotide variants, copy number alterations, and other insertions/deletions are linked to cfDNA/ctDNA (Ganesamoorthy et al., 2022). Using current DNA sequencing techniques, quantitative analyses of ctDNA can be performed using digital PCR, BEAMing, NGS, and ARMS after DNA amplification (Dono et al., 2019; Vanni et al., 2015).

### 2.2 Circulating tumor RNA (ctRNA)

Recently, researchers have shown that miRNA can be detected, quantified, and separated in a very stable state in cell-free components of body fluids, which makes it a potential circulating biomarker for various cancers (Ambros, 2004a). miRNAs are single-stranded, highly conserved RNA molecules that are 19–22 nucleotides long and target mRNA for gene regulation. Mature miRNA can cause RNA-induced silencing by forming base complementary pairs with target mRNA to inhibit translation or degrade the target mRNA (Ambros, 2004a). Much scientific evidence suggests that mutations or dysregulation of miRNAs could make them potentially oncogenic and directly affect several diseases, including cancer (Croce, 2009).

In plasma, ctRNA can be found as peripheral circulating nucleic acids. The extracellular RNAs (mRNA, miRNA, long non-coding RNA, and other types of RNA) are secreted through protein–mRNA complexes, exosomes, tumor-educated platelets, or apoptotic bodies (Figure 3B) (Croce, 2009). While exploring the potential use of liquid biopsy as a diagnostic tool for lung cancer, researchers suggested that serum or plasma levels of miRNAs could be potential biomarkers of early-stage NSCLC (Figure 2) (Zhang et al., 2019). For example, Let-7a, a biomarker found in low concentrations in NSCLC patients, has been linked to mutational changes such as single nucleotide polymorphisms at let-7 complementary sites (LCS) (Pop-Bica et al., 2020; Chin et al., 2008). This association was found in a study that sequenced 74 NSCLC tissue samples to detect mutations at LCS in the *KRAS* 3′ UTR (Chin et al., 2008). In serum, miR-1246 and miR-1290 levels were linked to the progression and metastasis of NSCLC, suggesting their potential as clinical biomarkers (Zhang et al., 2016).

Moreover, a wide scale analysis collating from several studies discovered five miRNAs (miR-21-5p, miR-155-5p, miR-223-3p, miR-126-3p and miR-221-3p), which could serve as potential biomarkers for lung cancer analysis and tumor biology (Tang et al., 2021; Di Paolo et al., 2021; Zhu et al., 2020; Zhu et al., 2022). Inhibiting miR-21 increased tumor cell sensitivity to gemcitabine, a chemotherapy drug (Meng et al., 2006). Those findings have led to speculation that miRNAs are actively involved in regulating tumor cell sensitivity to chemotherapeutic drugs.

### 2.3 Exosomes

Exosomes are small membranous vesicles derived from multistep endocytosis, typically 40–150 nm in diameter (with an average diameter of about 100 nm) (Kalluri and LeBleu, 2020; Urbanelli et al., 2013). Exosomes are present in various cell types and can be secreted into all body fluids (Figure 3C). They can transport DNA, mRNA, proteins, and lipids (Mao et al., 2021). Exosomes contain molecules that become activated through receptor-mediated binding, and they play a key role in regulating cell–cell communication, especially in tumor contexts (Xu et al., 2021). Thus, exosomes are great biomarkers for understanding lung cancer biology because they are involved in tumor growth, angiogenesis, epithelial–mesenchymal transition (EMT), invasion, and metastasis (Valadi et al., 2007). Their lipid bilayer structure prevents them from being degraded in high pH conditions or by ribonuclease activity (Valadi et al., 2007). Exosomes contribute to the maintenance of intracellular homeostasis by removing unnecessary cellular components (Mao et al., 2021). Thus, exosomes that are mutated and secreted by tumor cells could potentially have oncogenic properties. In fact, many researchers have discovered that exosomes play crucial roles in the development of lung cancer.

A study conducted by Tang et al. examined the content of mutated miRNAs secreted by exosomes in the A549 and NCI-H1299 human NSCLC cell lines. They found that miRNA-200b/c were associated with EMT invasion, a hallmark of exosomes in tumor progression (Tang et al., 2018). Similar studies have also suggested that exosomes could serve as biomarkers for the early diagnosis, prognosis, and treatment of tumors (Qin et al., 2017). Further studies have shown that plasma exosomes contain specific microRNAs, including miR-30a-3p, miR30e-3p, miR-181-5p, and miR-361-5p, that can be used for adenocarcinoma diagnosis (Cazzoli et al., 2013).

Like cfDNA, exosomal RNA can be used to efficiently detect *EGFR* mutations commonly found in NSCLC (Jouida et al., 2021). Janowska-Wieczorek et al. demonstrated that exosomes from platelets could promote metastasis and angiogenesis by transferring glycoprotein IIbIIIa (CD41) and inducing cyclinD2 in lung cancer surface cells (Janowska-Wieczorek et al., 2005). Furthermore, miR-660-5p was found at higher levels in plasma from NSCLC patients than in healthy individuals. miR-660-5p has also been linked to NSCLC tumorigenesis by targeting KLF9, a Kruppel-like factor involved in regulating cellular proliferation (Yongjian Qi and Zhang, 2019).

### 2.4 Circulating tumor cells

CTCs were first depicted by Dr. Ashworth in 1869 in the blood of a man with metastatic cancer (Ashworth, 1869). Metastatic cancer cells can detach from the primary site, invade the neighboring extracellular matrix, enter the bloodstream, and circulate in the vascular system until they extravasate and form secondary tumors (Figure 3D) (Wirtz et al., 2011). Thus, CTCs in plasma samples can be used as early biomarkers of cancer cells with aggressive behavior (Heidrich et al., 2023). Furthermore, the outcomes of patients with advanced stage cancers have been associated with CTCs (Cristofanilli et al., 2004; Cohen et al., 2006). In NSCLC patients, higher numbers of CTCs correlate with a more advanced stage of the disease (Krebs et al., 2011). Patients with higher CTC counts tend to have shorter OS times than those with lower CTC counts, suggesting that CTCs could be valuable markers for predicting survival times and guiding treatment decisions (Krebs et al., 2011). CTCs have been also proposed as surrogate markers for distant metastasis in lung cancer (Tanaka et al., 2009). Given its rapid tumor spread and high number of CTCs, SCLC is a suitable model for investigating the relationship between CTCs and CSCs (Hochmair et al., 2020). Hofman et al. used the Isolation by Size of Epithelial Tumor Cell method to harvest CTCs based on their size. When they applied this technique to 250 blood samples from NSCLC patients and 59 healthy controls, they detected malignant circulating non-hematological cells in 102 of the NSCLC patient samples (41%) and 0% of the healthy individuals (Hofman et al., 2012). Although diagnostic approaches using CTCs face challenges, such as the low cell counts in given blood samples and limited cell harvesting technologies (Lawrence et al., 2023), plasma CTCs have great potential as clinical biomarkers.

## 3 CSC-derived circulating factors

The study of CSCs began in 1994, when it was reported that CD34/CD38 cells were human acute myeloid leukemia stem cells (Lapidot et al., 1994). Recent research on CSCs has isolated them from almost all solid cancer populations (Najafi et al., 2019). CSCs have the characteristics of self-renewal, multidirectional differentiation, and unlimited proliferation, and they show resistance to chemotherapeutic drugs, strong tumorigenicity, and a strong ability to invade and metastasize (Najafi et al., 2019). Those findings have driven studies of lung-derived CSCs as potential lung cancer treatments (Kim et al., 2014). Many studies have been conducted to better understand the molecular basis of CSCs and their roles in tumor progression, therapeutic resistance, and metastasis (Najafi et al., 2019; Kim et al., 2014). Those studies have distinguished CSCs from the bulk of tumor cells through surface biomarkers (Singh and Settleman, 2010).

Several strategies are being used to develop anticancer drug treatments that specifically target CSCs (Singh and Settleman, 2010). Lung CSCs (LCSCs) and CSCs from various other tumor types can activate the TGF-β signaling pathway, which enhances cell migration capabilities (Du et al., 2015; Murai et al., 2015; Xu et al., 2009; Hao et al., 2019) and thereby affects the malignancy and clinical prognosis of tumors.

### 3.1 CD133-related biomarkers in CSCs

CD133, also known as AC133 and promini-1, is a 97-kDa pentaspan transmembrane glycoprotein (Glumac and LeBeau, 2018). CD133’s initial discovery in 1997 was as a hematopoietic stem cell (HSC) marker (Yin et al., 1997; Miraglia et al., 1997). In HSCs, the biological function of CD133 has been correlated with stem cell fate decisions and emerged as an important physiological regulator of stem cell maintenance and expansion (Kuci et al., 2003). CD133 is the cell surface antigen most frequently used to detect and isolate CSCs from various solid tumors, including those of the brain, colon, pancreas, prostate, lung, and liver (Kim and Ryu, 2017; Liu et al., 2015). CD133 has also been used in combination with other markers or on its own to identify stem cells from a variety of tissues (Corbeil et al., 2001; Su et al., 2015). These studies have indicated that CD133 has a potential role in determining cellular fate or maintaining stem cell-like properties (Bauer et al., 2011; Bauer et al., 2008), although the exact mechanisms are still unclear.

CD133 is a tumor marker commonly used to identify CSC populations in lung cancer (Kemper et al., 2010; Grosse-Gehling et al., 2013; Qu et al., 2013). CD133 has been postulated as a CSC marker in both NSCLC and SCLC (Wu and Wu, 2009). Most major studies of CD133 indicate that it has significant prognostic and predictive value for OS, disease-free survival (DFS), and progression-free survival (PFS) times in many different solid cancers (Grosse-Gehling et al., 2013). CSC-derived CD133 has been observed to affect many pathways in lung cancer, such as the P13K, ATK, Wnt, and JNK pathways (Zheng et al., 2022). The resistance of both CSCs and CD133^+^ cell fractions to chemotherapy and radiotherapy highlights their potential to predict patient responses to these treatments (Kemper et al., 2010; Grosse-Gehling et al., 2013; Chen et al., 2008; Blazek et al., 2007; Lin et al., 2010) and thereby address a significant unmet clinical need in the context of CD133 overexpression, which is associated with poor prognosis and reduced OS times in various cancers.

A well-known biomarker for CSCs is the combination of CD133^+^ and CD326^-^ in extracellular vesicles (EVs) (Brocco et al., 2019). Brocco et al. combined different biomarkers for tumor CSC-derived EVs and showed that the concentration of circulating tumor CSC–derived CD133^+^ CD326^-^ EVs was higher in lung cancer patients than in healthy volunteers (Brocco et al., 2019). This finding is supported by several other studies whose evidence indicates that tumor cells have a higher concentration of EVs than nonmalignant cells (Bebelman et al., 2018). The level of CD133^+^ CD326^-^ EVs has been suggested to play a role in patient longevity (Brocco et al., 2019). Overall, lung cancer patients with higher concentrations of CD133^+^ CD326^-^ EVs had poorer OS times than those with lower concentrations (Brocco et al., 2019). It has been observed that concentrations of CD133^+^ CD326^-^ EVs decrease when lung cancer patients respond to anticancer therapy (Brocco et al., 2019), and lower levels of circulating CD133^+^ CD336^-^ EVs in peripheral blood from lung cancer patients are associated with longer survival times compared with patients with higher levels (Brocco et al., 2019). The data thus highly suggest that circulating tumor CSC–derived CD133^+^ CD326^-^ EVs can be used as screening and diagnostic biomarkers for lung cancer and that patient clinical outcomes correlate with the concentration of CD133^+^ CD326^-^ EVs (Brocco et al., 2019) (Table 2). Thus, tumor stem cell–derived CD133^+^ CD326^-^ EVs circulating in peripheral blood could be used in liquid biopsies as biomarkers for early diagnosis and for monitoring the temporal and spatial heterogeneity of tumor cells.

**TABLE 2:** Circulating biomarkers in lung cancer stem cells.

Biomarker	Type	Affected/Targeted Genes	Reference
CSC-CD133^+^ CD326^-^ EV	CSC	*P13K*, *ATK*, *Wnt*, *JNK*	Zheng et al. (2022), Brocco et al. (2019)
CSC-miRNA-23a	CSC-Exos-miRNA	*PTEN*, *PI3K*, *AKT*, *STAT3*	Han et al. (2017), Hsu et al. (2017), Aramini et al. (2020)
CSC-miRNA-128	miRNA	*AKT*, *p38*, *ERK*, *P13K*	Jiang et al. (2016), Hu et al. (2014), Pan et al. (2018)
CSC-miRNA-486-5p	miRNA (CSCs^−^ CD133^+^)	*p85*, *PI3K*, *AKT*	Moro et al. (2022)

### 3.2 CSC-derived exosomes in cancer

CSC-derived exosomes (CSC-Exos) are membranous vesicles secreted by CSCs (Li X. et al., 2022a). CSC-Exos mediate the exchange of information and materials between cells, activating signaling pathways that further promote tumor development (Annett and Robson, 2018; Lopatina et al., 2016; Wortzel et al., 2019). CSC-Exo formation involves four processes: budding, invagination, multivesicular body formation, and secretion. The molecular cargo of CSC-Exos derives in part from the surface of the parent tumor cell (Zhou et al., 2021). CSC-Exos thus carry materials between cancer cells and normal cells, including oncogenes, lipids, and nucleic acids (Lopatina et al., 2016). CSCs-Exos can cause cancer metastasis and infiltration by transferring proteins, lipids, and RNA to recipient cells to weaken the adhesion between cells (Wortzel et al., 2019). When CSC-Exos reach a recipient cell, they release their cargo through methods such as ligand binding, phagocytosis, and fusion with the plasma membrane, thereby regulating gene expression in the recipient cell (Lopatina et al., 2016). Data (Wang and Zhang, 2021) also show that the cargo of CSC-Exos, such as miRNA and proteins, contributes to the evasion of immune surveillance. Therefore, CSC-Exos play important roles in the development of cancer and might serve as targets for cancer treatment (Yang et al., 2020; Whiteside, 2016).

### 3.3 CSC-exos in lung cancer

CSC-Exos are nanosized vesicles used to mediate communication between cancer cells and the EMT (Li X. et al., 2022a). They have been shown to regulate cancer cell proliferation and apoptosis, which are controlled by programed regulatory pathways (Neutzner et al., 2012) that are frequently disrupted in cancer cells by mutations of tumor regulators (both proto-oncogenes and tumor suppressor genes), leading to malignant cell proliferation (Neutzner et al., 2012; Kontomanolis et al., 2020). One of the main factors that causes tumor angiogenesis is oxygen deficiency, which can promote the expression of exosomal miRNA (Li X. et al., 2022a). A high concentration of circulating miRNA-23a is often found in lung cancer patients (Han et al., 2017). Under hypoxic conditions, lung cancer–derived exosomes carry miRNA-23a, which targets prolyl hydroxylases and the tight junction protein ZO-1, promoting angiogenesis and cancer progression (Hsu et al., 2017). miRNA-23a has been shown to inhibit the effectiveness of lung cancer drugs such as EGFR-targeted tyrosine kinase inhibitors by suppressing their ability to phosphorylate PI3K and AKT in lung CSCs, thereby preventing the induction of apoptosis in the cells (Han et al., 2017). An upregulation in the concentration of miRNA-23a is tied to the downregulation of *PTEN* in lung CSCs (Table 2).

Potent miRNA-128 downregulation in lung cancer patients correlates with tumor differentiation, pathological changes, and metastasis through the targeting of the P13K, ATK, and p38 signaling pathways (Jiang et al., 2016; Hu et al., 2014). Whole blood concentration levels of miRNA-128 in lung cancer patients were lower than those in healthy controls, indicating that the expression levels of miRNA-128 correlate negatively with lung cancer and could be used for the early detection of lung cancer (Pan et al., 2018) (Table 2). In gefitinib-resistant PC9-CSCs, the expression of miRNA-128 is markedly suppressed compared with non-CSCs, and miRNA-128 upregulation reverses gefitinib resistance by inhibiting the c-Met/P13K/AKT pathway (Hu et al., 2014).

Using exosomes for tumor monitoring has significant advantages (Kok and Yu, 2020) because they are widely present in a variety of bodily fluids, and they are more specific than traditional tumor markers (Kok and Yu, 2020; Panigrahi and Deep, 2017). Detecting exosomes in bodily fluids such as urine or blood would enable changes in molecular markers to be tracked over time during disease development, and their presence in bodily fluids makes exosome markers easy to monitor and collect (Kok and Yu, 2020). Studies have indicated that the contents by exosomes released into body fluids, such as miRNA and proteins, have a different composition in patients with lung cancer than they do in healthy individuals (Dejima et al., 2017). Therefore, the contents of exosomes can be used as specific markers for certain tumors (Li X. et al., 2022a; Kok and Yu, 2020). An understanding of the role of CSC-Exos and their mechanisms could be used to effectively block related signaling pathways in cancer treatment (Li X. et al., 2022a).

### 3.4 miRNA in cancer

miRNA is one of the most promising molecular markers for tumor diagnosis and prognosis (Leng et al., 2017; Zheng et al., 2013). The main functions of miRNAs include sequence-specific target recognition and the regulation of gene expression at the post-transcriptional level (Leng et al., 2017; Zheng et al., 2013). miRNAs also significantly affect the gene expression and cellular signaling pathways in recipient cells by delivering their payload and thereby maintaining a dynamic equilibrium between CSCs and non-CSCs (Aramini et al., 2020). miRNAs have been associated with a variety of intracellular mechanisms, such as chemo- and radio-resistance, tumor recurrence, tumor growth, tumor metabolic alterations, and proliferation (Aramini et al., 2020; Pugh and Ratcliffe, 2017). The prognostic value of miRNA as both a qualitative and quantitative biomarker in plasma, in either its free from or encapsulated in macrovesicles, has been documented in various cancers (Zheng et al., 2013). Because miRNAs play a pivotal role in the physiological functioning of a cell, their dysregulation correlates with various pathologies, including the initiation and progression of cancer (Aramini et al., 2020; Zheng et al., 2013).

### 3.5 miRNAs as biomarkers for lung cancer

Dejima et al. (2017) demonstrated that patients with lung cancer have a significant upregulation of plasma exosomal miRNAs, including miRNA-21 and miRNA-4257, compared with healthy individuals. Their research data further show a correlation between the levels of exosomal miRNA-21/miRNA-4257 and indications of malignancy, such as tumor growth and metastatic invasion, in lung cancer (Dejima et al., 2017). Cazzoli et al. (2013) showed how exosomal miRNAs can be used to distinguish lung cancer patients from healthy individuals. They used 30 plasma samples: 10 from lung granuloma patients, 10 from lung adenocarcinoma patients, and 10 from matched healthy smokers. As shown by those studies, exosomal miRNAs hold promise as biomarkers for identifying and monitoring lung cancer in patients.

### 3.6 miRNAs as biomarkers in lung cancer stem cells

Exosomal miRNA-23a is a biomarker present in hypoxic lung cancer cells. It can enhance vessel membrane permeability and increase vascularization through STAT3 mechanisms, leading to the transformation of normal bronchial cells into malignant ones (Aramini et al., 2020). Another CSC biomarker is miRNA-486-5p, which is used to target CD133^+^ LCSC through the p85 and ATK pathways (Moro et al., 2022). miRNA-486-5p is a tumor suppressor that is downregulated in lung cancer patients, compared with normal lung tissue (Moro et al., 2022). Overexpression of miRNA-486-5p in CD133^+^ lung CSCs reduces the number of CD133^+^ CSCs by inhibiting their PI3K and AKT survival pathways (Moro et al., 2022). Thus, the potential biomarker miRNA-486-5p could be used to detect lung cancer in its early stages (Moro et al., 2022) (Table 2). Interestingly, miRNA-486-5p acts as an oncogenic miRNA by targeting the tumor suppressors *PTEN* and *FOXO1* in human glioblastoma multiforme (GBM) spheroids (Lopez-Bertoni et al., 2020). The SOX2 transcription factor, a protein important for maintaining the self-renewal properties of neural stem cells, binds to the promoter region of miRNA-486-5p for activation in GBM cells, eventually leading to stemness induction and therapy resistance (Lopez-Bertoni et al., 2020). Those results indicate that miRNA-486-5p might have different functional roles in tumorigenesis depending on the cellular context.

Lung cancer cell–derived exosomal miRNAs are interesting to researchers for their roles as biomarkers: predictors of recurrence in early-stage cases and prognostic indicators in treated patients with advanced disease (Tai et al., 2018; Liu et al., 2017). Because studies have demonstrated that exosomal miRNAs play a crucial role in metastatic processes and that they are present in a variety of human biofluids, they are being seen as potential biomarkers for future personalized medicine applications (Iliescu et al., 2019). Exosomal miRNAs are already being used as reliable biomarkers for detecting and diagnosing lung cancer in patients (Cazzoli et al., 2013; Rabinowits et al., 2009). The purpose of current studies is to explore the possibilities of using miRNAs as potential biomarkers for both early diagnosis of lung cancer and targeted therapy (Reclusa et al., 2017; Taverna et al., 2016).

## 4 Circulating biomarkers in advanced NSCLC

### 4.1 Circulating tumor DNA in advanced lung cancer

**FIGURE 4:**
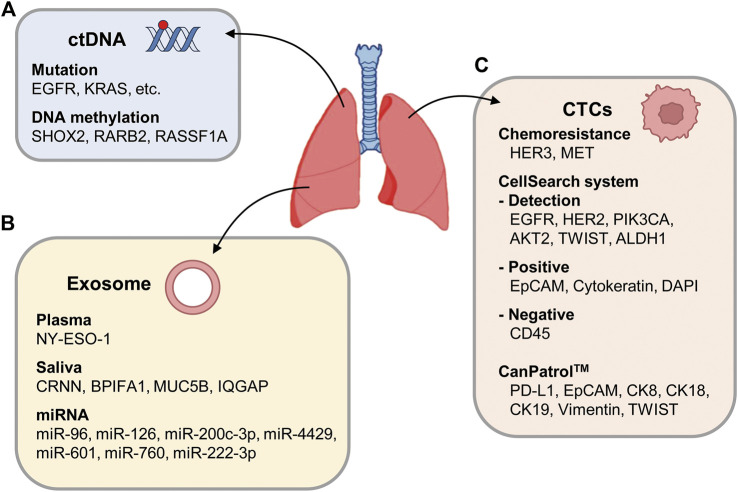
Schematic representation of circulating factors. **(A)** ctDNA may carry genetic mutation or DNA methylation changes in genes such as *SHOX2*, *RARB2*, and *RASSF1A.* **(B)** Exosomes are secreted from cancer cells, transporting miRNA or ctDNA into blood vessels. **(C)** Representative CTC markers of advanced NSCLC including *EGFR*, *HER2*, *PIK3CA*, *AKT2*, *TWIST*, and *ALDH1* with the CellSearch system. In the CellSearch system, a CTC is characterized as positivity for EpCAM, cytokeratins, and DAPI and negativity for CD45. In subpopulations of NSCLC patients with chemoresistance, *HER3* and *MET* are expressed in CTCs. As biomarkers of CTCs, PD-L1, EpCAM, CK8, CK18, CK19, vimentin, and TWIST expressions were also tested.

ctDNA is a valuable, non-invasive diagnostic and prognostic biomarker for advanced cancer (Figure 4A). ctDNA can be identified as cfDNA with oncogenic mutations. A recent clinicopathological analysis showed that the OS and PFS times of NSCLC patients with high cfDNA concentrations (median PFS = 5.6 months, median OS = 10.4 months) were shorter than those with low cfDNA concentrations (median PFS = 15.8 months, median OS = 28.9 months) (Hyun et al., 2017).

EGFR mutations are a standard prognostic biomarker when treating patients with advanced NSCLC with first-line EGFR tyrosine-kinase inhibitors (Mok et al., 2015; Zhang et al., 2018). EGFR mutations in plasma ctDNA were detected using an amplification refractory mutation system, and mutations were found in 251 of 1001 patients (25.1%). EGFR mutations were found in 26.8% of lung adenocarcinoma (LUAD) patients (n = 868) and 11.7% of lung squamous cell carcinoma patients (n = 120) (Zhang et al., 2018). To detect ctDNA in advanced NSCLC patients (stages II–IV), cancer personalized profiling by deep sequencing was introduced and detected ctDNA in 100% of patients with stage II–IV NSCLC and 50% of patients with stage 1 NSCLC (Newman et al., 2014). To identify genes that harbored recurrent mutations, the cancer genome sequencing data were compared with data for NSCLC patients in the Cancer Genome Atlas. Using that information, complementary hybridization was performed with a designed DNA capture reagent. Capturing ctDNA in blood samples from NSCLC patients and performing deep sequencing can be useful for diagnosing advanced NSCLC and monitoring tumor progression (Newman et al., 2014; Spellman and Gray, 2014). A study using TRACERx (tracking non-small-cell lung cancer evolution through therapy) for ctDNA profiling in NSCLC patients (Abbosh et al., 2017) showed that ctDNA profiling can track the subclonal traits of NSCLC metastasis and relapse. In another study, ctDNA from CTCs was extracted from metastatic NSCLC patients to detect the presence of KRAS mutations using digital droplet PCR (Guibert et al., 2016). That study found KRAS mutations in 82% of patients when ctDNA was used and in 34% of patients when CTCs were used. The presence of KRAS mutation in ctDNA from CTCs correlated with a poor response to chemotherapy or targeted therapy. Monitoring KRAS-mutated-DNA in cfDNA correlated with the treatment response (87.5%) more highly than monitoring KRAS-mutated-DNA in CTCs (37.5%) (Guibert et al., 2016). Analyzing liquid biopsies with digital droplet PCR thus showed promise in predicting and monitoring treatment responses in KRAS-mutated LUAD (Figure 4A). Since KRAS mutations are typically associated with resistance to EGFR TKIs, detecting KRAS mutations in liquid biopsy samples allows for more targeted patient treatment (Eberhard et al., 2005; Pao et al., 2005).

To increase the detection of early stage of NSCLC, DNA methylation was investigated. Methylated ctDNA is more easily detected in the early stage of NSCLC because DNA methylation occurs in the early stage of carcinogenesis (Diaz and Bardelli, 2014). DNA methylation of *SHOX2* is a biomarker for lung cancer diagnosis. The levels of methylated ctDNA in plasma from stage I patients were significantly lower than those in plasma from patients with advanced-stage disease (Kneip et al., 2011), indicating that methylated ctDNA is more abundant in advanced cancer. Using quantitative methylation-specific PCR, the index of methylation (IM) can be calculated (Ponomaryova et al., 2013). In advanced stages of NSCLC, high IM values can be observed, especially in the *RARB2* gene, though not in the *RASSF1A* gene. A multivariate analysis of variance revealed that the IM of two genes from ctDNA was highly associated with a lung cancer diagnosis in advanced NSCLC, indicating the significance of this technique (Ponomaryova et al., 2013). Thus, abnormal methylation of ctDNA is also a valuable tool to increase the sensitivity of diagnosis and prognosis in advanced NSCLC.

### 4.2 Exosomes in advanced NSCLC

Exosomal biomarkers in plasma or saliva from cancer patients were analyzed to diagnose and predict the survival rates of NSCLC patients (Sun et al., 2018; Sandfeld-Paulsen et al., 2016). The exosomes in plasma from 276 NSCLC patients were phenotyped using an extracellular vesicle array. Forty-nine antibodies captured the proteins on the exosomes, and NY-ESO-1 was suggested as a possible marker with a significant correlation with the survival rate, depending on the concentration (Table 1; Figure 4B). In the saliva of NSCLC cancer patients, CRNN, BPIFA1, MUC5B, and IQGAP, which were detected in a proteomic analysis, differed significantly from the control group (Sun et al., 2018) (Table 1). Because they do not require invasive collection from patients, the salivary exosomal biomarkers can easily be applied to diagnose cancer patients. An analysis of exosome biomarkers in serum from healthy people and patients with early- and late-stage lung cancer revealed that vimentin expression was higher in late-stage patients than in healthy people (Rahman et al., 2016), indicating that the EMT marker could be used to identify advanced cancer (Table 1; Figure 4B).

Exosomal miRNA differs significantly from total exosomes in lung cancer (Rabinowits et al., 2009). miRNA and total exosomes were compared between 27 patients with NSCLC, specifically LUAD, and nine controls (aged 21–80 years). The mean exosome concentrations in the LUAD and control groups were 2.85 mg/mL (95% CI, 1.94–3.76) and 0.77 mg/mL (95% CI, 0.68–0.86), respectively (*p* < 0.001) (Rabinowits et al., 2009), and the mean miRNA concentrations were 0.1586 mg/mL (95% CI, 145.7–171.5) and 0.0681 mg/mL (95% CI, 57.2–78.9), respectively (*p* < 0.001) (Rabinowits et al., 2009), indicating that the concentration of total exosome is much higher than that of exosomal miRNA. To identify miRNA biomarkers in exosomes, exosomes were isolated from the serum of 45 NSCLC patients classified according to the TNM stage (Grimolizzi et al., 2017). High levels of miRNA126 were detected in advanced stages of patients compared with healthy controls (Grimolizzi et al., 2017) (Table 1). Additional studies revealed that exosomal miR-96 was highly expressed in the serums and tissues of lung cancer patients with advanced disease (grades II to IV). In serum and tissues from patients with lymph node metastasis, the expression of exosomal miR-96 was about two times higher than in those without metastasis (Wu et al., 2017) (Table 1). These data suggest that exosomal miR-126 and miR-96 could be used as circulating biomarkers indicating advanced NSCLC. A recent study analyzed miRNA profiles of plasma-derived exosomes from healthy individuals, primary NSCLC patients, and metastatic NSCLC patients (Geng et al., 2023), and reported that miR-200c-3p and miR-4429 were remarkedly upregulated in metastatic NSCLC patients (Table 1; Figure 4B). Moreover, serum miR-601 and miR-760 were suggested as potential biomarkers for NSCLC patients, following qRT-PCR experiments with 70 patients (El-Aal et al., 2023).

During cancer treatment, chemoresistance occurs frequently. To understand chemoresistance-related miRNA, samples from NSCLC patients treated with chemotherapies were analyzed (Wei et al., 2017). A clinical analysis of NSCLC patients treated with gemcitabine revealed that higher levels of exosomal miR-222-3p correlated with a poor prognosis after gemcitabine treatment (Wei et al., 2017) (Table 1; Figure 4B). In addition, miR-96 levels correlated with cisplatin-resistance (Wu et al., 2017). Therefore, exosomal miRNA in NSCLC patients is also suitable for predicting sensitivity to specific anticancer drugs.

### 4.3 CTCs analysis in advanced NSCLC

CTCs, epithelial cells detached from tumors, are a promising biomarker for cancer diagnosis because they can be found in the blood of advanced NSCLC patients with a frequency of 1 CTC per 10^6^–10^7^ leukocytes, and they can aggregate to form tumor microemboli (Tartarone et al., 2017). RT-PCR can be used to detect CTCs in the peripheral blood of patients, and cell surface markers can be used to increase sensitivity (Hanssen et al., 2016) (Figure 4C). Although the epithelial cell adhesion molecule (EpCAM) is useful as a CTC marker, EpCAM-positive CTCs are found in NSCLC patients less frequently than in other epithelial tumors because of the EMT that occurs in advanced NSCLC. In the advanced phases of NSCLC, *EGFR*, *HER2*, *PIK3CA*, *AKT2*, *TWIST*, and *ALDH1* can be used together to detect CTCs with the CellSearch system, a dominant detection method for CTCs, and multiplex RT-PCR methods. In the CellSearch system, a CTC is characterized as positivity for EpCAM, cytokeratins, and DAPI and negativity for CD45. In NSCLC, 86% of CTC-positive patients were found to express ALDH1 (Hanssen et al., 2016). Of note, NSCLC patients with an altered *EGFR* genotype had CTC-positive cells more frequently than those with wildtype *EGFR*. In subpopulations of NSCLC patients with chemoresistance, *HER3* and *MET* are expressed in CTCs.

The CanPatrol™ (Surexam Biotech, China) CTC enrichment technique was applied to detect mesenchymal CTCs and prognostic biomarkers in the pulmonary veins of 114 NSCLC patients in stages I–III (Dong et al., 2019). CTCs can be classified into three types depending on the analysis method. E-CTCs have epithelial markers without mesenchymal markers, M-CTCs have mesenchymal markers without epithelial markers, and hybrid E/M-CTCs have both epithelial and mesenchymal markers (Zhang et al., 2022). The CanPatrol system detected CTCs in 96.5% of patients. As biomarkers of CTCs, PD-L1, EpCAM, CK8, CK18, CK19, vimentin, and TWIST expressions were also tested with *in situ* hybridization. When the clinical relevance of being double negative for *EGFR* and *ALK* was analyzed, OS and DFS times were reduced in PD-L1 (+) CTC patients (Dong et al., 2019). An additional mesenchymal CTC analysis revealed that DFS was shorter in patients with mesenchymal CTC biomarkers than in those with epithelial markers. High overall numbers of CTCs, mesenchymal CTCs, and PD-L1(+) CTCs all correlated with short DFS times in NSCLC patients (Dong et al., 2019). Using the CTCs biomarkers mentioned above, several sensitive detection methods have been developed as diagnostics and prognostics for cancer patients.

## 5 Clinical studies with circulating factors

Clinical studies investigating circulating factors such as CTCs, ctDNA, miRNA, and exosomes in lung cancer patients are currently underway. ctDNA has garnered particular attention, resulting in a wealth of published clinical reports and data. However, research involving the other circulating factors is also gaining momentum, with various clinical studies exploring their roles and significance in lung cancer management. All these studies aim to shed light on the clinical utility and potential of CTCs, ctDNA, miRNA, and exosomes in improving the diagnosis, prognosis, and treatment of lung cancer (Figure 2).

One clinical study evaluated the use of ctDNA and CTCs for predicting lung cancer activity and drug response in lung cancer patients (NCT04254497). That study compared the prognostic importance of ctDNA and CTCs detected using ultrasensitive amplicon-based NGS with a tissue *EGFR* test. Using NGS, ctDNA was detected in blood samples from 98.1% of patients, whereas the tissue EGFR test detected ctDNA in only 70% of the samples (Choudhury et al., 2022a). Adding plasma NGS to tissue EGFR testing thus increased the detection sensitivity (Choudhury et al., 2022a). EGFR is a significant causal factor in lung cancer development, and its inhibitors have proved effective in treating lung cancer. In a clinical study (NCT01734915), researchers used a noninvasive CTC and ctDNA analysis to detect the EGFR T790M mutation in NSCLC patients and predict their response to T790M-targeted EGFR inhibitors (Sundaresan et al., 2016). Even though the individual CTC and ctDNA analyses were successful in only 20%–30% of cases, when they were used together, they enabled genotyping in all patients with an available blood sample. This combined approach identified the T790M mutation in 35% patients, even when concurrent biopsy results were negative or inconclusive (Sundaresan et al., 2016).

In a study of 99 patients with primary lung cancer (NCT03479099), the diagnostic significance of ctDNA was evaluated by using NGS to analyze single nucleotide variants (Table 3), with *TP53*, *CDKN2A*, and *EGFR* being found the most frequently (Moon et al., 2020). The diagnostic sensitivities of a conventional tumor marker (combination of carcinoembryonic antigen/CYFRA 21-1/neuron-specific enolase), ctDNA, and CTCs were 66.7%, 72.7%, and 65.7%, respectively (Moon et al., 2020), indicating that ctDNA is the most sensitive marker for lung cancer. Another comprehensive molecular profiling study (NCT03512847) involved metastatic NSCLC patients treated with immunotherapy or chemotherapy to explore the role of ctDNA as a biomarker and the potential of its molecular profiles to predict the responses to those treatments (Table 3). Among 51 patients with advanced NSCLC, 46 had sufficient material for PD-L1 analyses, including the PD-L1 tumor proportion score (TPS) (Frank et al., 2020). Clinically relevant changes in the PD-L1 TPS were observed in 17% of the advanced NSCLC patients, and the PD-L1 TPS was found to be much higher in patients treated with chemotherapy than in those treated with immunotherapy (Frank et al., 2020). These findings offer valuable insights into the potential use of ctDNA as a biomarker that can guide treatment decisions for NSCLC patients.

**TABLE 3:** Current clinical trials with circulating factors in NSCLC patients.

NCT registry number	Targets	Phase	No. of Patients	Method	Status	Ref.
NCT03512847	NSCLC ctDNA	—	150	Predictive gene profiles and dynamic measurement of treatment response	Completed	Frank et al. (2020)
NCT03465241	NSCLC ctDNA	IIA-IIIA	119	ctDNA dynamic monitoring	Completed	Li et al. (2022b)
NCT03479099	Lung cancer (Diagnosis) CTC ctDNA	I	111	Clinical Utility of Combined CTC and ctDNA Assay	Completed	Moon et al. (2020)
NCT05441566	NSCLC MRD ctDNA	IB-IIA	60	Dynamic monitoring driver gene	Enrolling	-
NCT04966663	NSCLC Complete surgical resection ctDNA	II	66	ctDNA blood test w/Nivolumab, Pemetrexed, Gemcitabine, Cisplatin, Carboplatin	Recruiting	-
NCT05815407	NSCLC miRNA 106b-5p, 601, 760	Early stage	100	qRT-PCR expression profiling of the selected miRNAs	Completed	El-Aal et al. (2023)
NCT05854030	Lung cancer ExosomealmiRNA	—	60	Diagnostic Test, collect plasma samples and clinical features	Active	-
NCT04939324	NSCLC Exosomes	—	30	Diagnostic Test, blood samples	Active	-
NCT04529915	NSCLC Exosomes	—	470	Diagnostic Test, blood plasma	Active	-
NCT04629079	Lung cancer exosomes P4HA1	—	800	Blood Exosomes and HRCT	Active	-

A prospective study was performed in 123 patients with resectable stage I to III NSCLC to understand perioperative ctDNA in patients with operable NSCLC (NCT03465241) (Table 3). In 24.8% of the patients analyzed, presurgical ctDNA was detectable, and it was associated with shorter recurrence-free survival and OS times (Li N. et al., 2022b). Similarly, postsurgical ctDNA was observed in 10.3% of patients and correlated with shorter survival times (Li N. et al., 2022b). These findings suggest that perioperative ctDNA analyses can predict the cancer recurrence and survival rates of NSCLC patients. Another clinical study (NCT02965391) investigated the half-life of ctDNA after surgery. The time scheme was 3 months and 3 years after surgery to see whether variations in ctDNA levels after surgery correlated with clinical features or tumor recurrence (Chen et al., 2019). The results show that the plasma ctDNA concentration decreased rapidly after radical tumor resection, with a median half-life of 35 min, indicating that ctDNA degrades after surgery. In addition, the detection of ctDNA 3 days after surgery was associated with the DFS times of patients (Chen et al., 2019), suggesting that ctDNA detection can help to plan precise individual treatment and follow-up for NSCLC patients after surgery. Recently, clinical trials have been opened to investigate clinical values associated with recurrence in the advanced stages of NSCLC (NCT05441566), to understand whether adjuvant treatment can help reduce the recurrence risk associated with ctDNA in the blood (NCT04966663), and so on. Upon the completion of those clinical trials, the use of ctDNA as a marker is expected to significantly improve the diagnosis and prognosis of NSCLC because it offers high sensitivity in detecting potential disease recurrence. These advances have the potential to enhance patient outcomes and guide the development of more effective and personalized treatment strategies for individuals with NSCLC.

Although clinical trials testing RNA are less frequent than those testing ctDNA, clinical tests such as NCT05815407 were performed to determine the prognostic or diagnostic potential of miRNAs (106b-5p, 601, and 760) in serum from NSCLC patients. miR601 and miR760 expression was downregulated in NSCLC, and the expression of whole miR-106b-5p was upregulated. Based on those miRNA expression patterns, miR-601 and miR-760 were suggested as potential diagnostic and prognostic biomarkers, respectively, for early-stage NSCLC patients (El-Aal et al., 2023).

Currently, several clinical studies are underway to use exosomes (NCT04939324, NCT04529915, NCT04629079), miRNA (NCT05854030), or ctDNA (NCT05441566, NCT04966663) as potential biomarkers, develop detection methods, or predict therapeutic efficacy in lung cancer. Although it hinges on the clinical data, diagnostic techniques, therapeutic efficacy, and survival predictions for patients are expected to improve.

## 6 Approved diagnostic assays for circulating factors

The integration of circulating biomarkers into clinical practice and the development of diagnostic tools for precisely detecting circulating factors have become increasingly significant, as they can lead to early detection, targeted therapy, and monitoring of patient response. The diagnostic assay tools approved to detect abnormalities in CTCs, ctDNA/cfDNA, or ctRNA include the Agilent Resolution ctDx FIRST Assay, cobas EGFR Mutation Test v2, Foundation One Assay, Guardant 360 Assay, and LiquidHALLMARK test kits (Table 4). The Agilent Resolution ctDx FIRST (Resolution Bioscience, Inc.) is a targeted NGS assay designed to detect a specific mutation in the *KRAS* gene within cfDNA in plasma samples and thereby help to identify NSCLC patients with certain gene mutations (Pekker et al., 2022). In a study by Pekker et al. (2022), in 2022, the Agilent Resolution ctDx FIRST successfully identified *KRAS* G12C variants from collected NSCLC plasma samples without showing any false positives in samples from healthy donors. A study led by Paweletz et al. (2016) used the Resolution Bioscience ctDx Lung Panel targeting mutations and rearrangements in NSCLC to test plasma cfDNA from 48 advanced NSCLC patients. They successfully detected genomic alterations specific to NSCLC, including *ALK*, *ROS1* and *RET* rearrangements, without false positives, demonstrating the test’s broad clinical potential (Paweletz et al., 2016). Mondaca et al. (2021) tested 736 plasma samples with the Resolution ctDNA NGS and identified *ALK* fusion in 21 patient samples. They also discovered new *ALK* fusion partners (*EML4*, *CLTC*, and *PON1S*) and compared their findings with results from tissue biopsies, showing a concordance rate of 93%. This suggests that the Resolution NGS tool can be used to detect ALK fusions in lung cancer patients (Mondaca et al., 2021).

**TABLE 4:** Diagnostic tools for circulating biomarkers in NSCLC.

Diagnostic Tool	Biomarker detected	Target	Method	Reference
Agilent Resolution ctDx FIRST Assay	cfDNA	*ALK, KRAS*, *RET*, *ROS1*	Next-Generation Sequencing	Pekker et al. (2022), Paweletz et al. (2016), Mondaca et al. (2021)
Cobas EGFR Mutation Test v2	cfDNA	*EGFR*	qRT-PCR	Malapelle et al. (2017), Szpechcinski et al. (2021)
FoundationOne Liquid CDx assay	cfDNA	*EGFR*	Next-Generation Sequencing	Woodhouse et al. (2020)
Guardant 360^®^ CDx (plasma) assay	ctDNA, cfRNA	*ALK*, *KRAS*	Next-Generation Sequencing	Bauml et al. (2022), Kwon et al. (2022)
LiquidHALLMARK^®^ Test	ctDNA, ctRNA	*ALK*, *EGFR*	Next-Generation Sequencing	Choudhury et al. (2022b), Poh et al. (2022)

The cobas EGFR Mutation Test v2 (Roche Molecular Systems, Inc.) is a real-time PCR assay designed to detect specific mutations of the *EGFR* gene in cfDNA from NSCLC patients (Malapelle et al., 2017; Szpechcinski et al., 2021). This assay can detect exon 19 deletions, the L858R mutation, and the T790M mutation in the *EGFR* gene. Szpechcinski et al. used the cobas EGFR Mutation Test v2 to analyze *EGFR* mutations in cfDNA from NSCLC plasma samples (Szpechcinski et al., 2021). They evaluated two real-time PCR-based methods, the cobas EGFR Mutation Test v2 and the therascreen EGFR Plasma RGQ PCR (Qiagen), and compared their accuracy in detecting *EGFR* mutations. They first collected blood samples from NSCLC patients at various stages, primarily to identify patients who might benefit from EGFR tyrosine kinase inhibitor therapy. Their results revealed that the cobas instrument was superior to the therascreen test in detecting plasma EGFR mutations in advanced NSCLC, with concordance rates of 90% and 73.33%, respectively. In addition, among patients with clinical progression on EGFR tyrosine kinase inhibitors, the cobas assay detected the T790M mutation in 30% of the plasma samples, compared with 23% with the therascreen PCR method (Szpechcinski et al., 2021).

The Foundation One Liquid CDx liquid biopsy test (Foundation Medicine, Inc.) is based on NGS technology. This assay can identify various genetic mutations, including *EGFR* exon 19 deletions and *EGFR* exon 21 L858R alterations, from cfDNA found in the plasma of cancer patients (Woodhouse et al., 2020). The authors conducted concordance studies for *EGFR* exon 19 deletions and exon 21 L858R mutations, comparing the results of the Foundation One Liquid CDx with those from the cobas EGFR Mutation Test v2. Their studies demonstrated non-inferior concordance, with the 177 NSCLS plasma samples showing more than 95% positive and negative agreement. This validation indicates that specific genetic alterations in NSCLC patients can be reliably identified using Foundation One Liquid CDx, enabling personalized therapy with EGFR tyrosine kinase inhibitors such as erlotinib, gefitinib, and osimertinib (Woodhouse et al., 2020).

Guardant360 CDx (Guardant Health, Inc.), also based on NGS, can detect abnormalities in NSCLC, including *ALK* rearrangement and *KRAS* p.G12C (Bauml et al., 2022; Kwon et al., 2022). Kwon et al. (2022) successfully demonstrated that the Guardant360 can detect *ALK* rearrangements and therapy-resistance mutations in cfDNA in plasma samples from lung cancer patients. The authors used the Guardant360 CDx assay to test plasma samples from 92 NSCLC patients pretreated with ALK tyrosine kinase inhibitors, and they identified *ALK* mutations known to cause resistance to ALK tyrosine kinase inhibitors, including L1196M, G1269A, and T1151R (Kwon et al., 2022). Bauml et al. (2022) also used Guardant360 CDx liquid biopsy analyses to detect *KRAS* p.G12C mutations in collected plasma samples. Their concordance studies, involving 189 patients, showed an overall agreement of 95%, strongly suggesting that Guardant360 CDx can be used to detect *KRAS* p.G12C mutations in NSCLC patients (Bauml et al., 2022). Sotorasib, a synthetic chemical compound developed by Amgen Inc., covalently binds to the KRAS^G12C^ mutant protein for inhibition, making it an effective treatment for NSCLC patients with this mutation (Hong et al., 2020).

The LiquidHALLMARK test (Lucence, Palo Alto, CA) is also an NGS-based assay. Choudhury et al. (2022b) used LiquidHALLMARK to examine plasma samples from patients with lung cancer and compared the results with those from tissue biopsies. They found that combining plasma testing with tissue EGFR significantly increased the mutation detection rates. They also demonstrated that the plasma test has a significantly shorter average turnaround time (10 days) than the conventional tissue testing approach (29.9 days). Poh et al. (2022) conducted analytical validation studies for LiquidHALLMARK with various plasma samples. They tested 355 lung cancer specimens for EGFR mutations with both LiquidHALLMARK and allele-specific PCR, showing an overall concordance of 93.8%. They also compared the results for 50 lung cancer samples with the cobas EGFR Mutation Test v2 and demonstrated a concordance of 84%. In addition, they showed a high detection rate of 74.8% for ctDNA in the cancer samples, affirming LiquidHALLMARK’s high sensitivity and endorsing its clinical utility as a plasma-based biomarker assay (Poh et al., 2022).

The diagnostic assays discussed here have made significant contributions to the detection of specific genetic mutations in circulating biomarkers associated with lung cancer. These tools have demonstrated clinical applications and enable personalized therapy based on patients’ molecular profiles. By providing real-time, non-invasive diagnostic tools, these assays may lead to precise treatment decisions and improved patient outcomes.

Ongoing research and development in the field of liquid biopsy will continue to uncover additional biomarkers, enhancing our understanding of lung cancer and other human diseases. The role of liquid biopsies in clinical practice will continue to expand, enabling more personalized and targeted therapies.

## 7 Clinical significance, advantages, and challenges

Understanding the clinical relevance of circulating biomarkers is crucial for their application in personalized therapies and patient care. Here, we provide an overview of the prognostic value, diagnostic accuracy, and diagnostic sensitivity of these biomarkers, including ctDNA, CTCs, exosomes, and microRNAs. Additionally, we discuss their advantages, limitations, and the major challenges associated with their clinical use.

### 7.1 ctDNA

Several next-generation sequencing assays have been employed to detect ctDNA in clinical samples. Various research teams have evaluated the assays by determining prognostic value, diagnostic accuracy, and sensitivity, for supporting accurate treatment decisions and detection of disease progression. Abbosh et al. (2017) used the NGS HiSeq 2000 (Illumina) and Ion S5 System (ThermoFisher Scientific), to conduct ctDNA profiling and accurately predict lung cancer recurrence risk. In their study of 96 patients with early-stage NSCLC, they analyzed single nucleotide variants (SNVs) and found that ctDNA detected at least two SNVs in 48% of cases and a single SNV in 12 additional cases. The study reported a tissue concordance rate of 61%, with a sensitivity of 99% and specificity of 99.6%. Additionally, their pathology data revealed that ctDNA detection was associated with different histological subtypes of lung cancer. In another study using the HiSeq 2500 (Illumina) with a custom TecSeq panel, ctDNA showed a tissue concordance of 72%, sensitivity ranging from 89% to 97.4%, and specificity of 100% (Phallen et al., 2017). In their study with 100 patients, 72% of the 216 detected alterations were identical in both plasma and tumor samples. Concordance was 77% among stage III and IV patients and 68% among early-stage patients.

Advantages of using ctDNA as a marker for NSCLC include its ability to accurately predict clinical responses to both first- and second-generation tyrosine kinase inhibitors (Ebert et al., 2020; Han et al., 2022). Furthermore, changes in plasma ctDNA levels can help determine the optimal timing for surgery in NSCLC patients (Zhao et al., 2023).

Major challenges include the low sensitivity in early-stage NSCLC patients due to the minimal amount of DNA released. The lack of standardized protocols for ctDNA extraction, analysis, and data interpretation complicates the integration of ctDNA detection into universal clinical diagnostic procedures. Additionally, the high cost of ctDNA detection equipment also poses a barrier to its global adoption (Zhu et al., 2024).

### 7.2 CTCs

The folate receptor is often overexpressed in various types of cancer cells, and the detection of folate receptor-positive circulating tumor cells (FR + -CTCs) in blood samples may provide insights into the progression of NSCLC and response to treatment (Wang et al., 2017; Yu et al., 2013). In evaluating clinical utility, two studies showed that FR + -CTC levels were significantly higher in lung cancer patients compared to normal controls. In one study, the FR + -CTC levels of 153 NSCLC patients were significantly higher than those of 49 healthy donors and 64 patients with benign lung diseases. The authors demonstrated a sensitivity of 73.2% and a specificity of 84.1% in diagnosing NSCLC, with a sensitivity of 67.2% for stage I disease (Yu et al., 2013). In another study, the authors detected FR + -CTCs using a novel ligand-targeted polymerase chain reaction (LT-PCR) detection technique and reported that FR + -CTC levels were significantly higher in lung cancer patients, with a sensitivity of 77.7% and a specificity of 89.5% for diagnosing lung cancer (Wang et al., 2017).

Another research team developed the L-MISC (Lung-Metastasis Initiating Stem Cells) nanosensor and identified unique metastatic signatures, even from a 5 μL blood sample, with a sensitivity of 100% and a specificity of 88% for distinguishing lung cancer patients from healthy individuals. Additionally, their machine-learning model predicted lung cancer metastasis with 100% sensitivity and specificity, allowing for accurate diagnosis of both primary and metastatic lung cancer (Premachandran et al., 2024).

Janning et al. (2019) demonstrated that there is a significant variation in the expression levels of PD-L1 among the CTCs obtained from NSCLC patients, reflecting the diverse nature of the tumor cells circulating in the bloodstream. This heterogeneity is considered as advantage of using CTCs as a marker for NSCLC, as it may allow for more accurate prediction of patients’ responses to PD-L1 targeted therapies. CTCs can also provide a reproductible indicator, and the positive detection rate of CTCs is higher than other tumor markers in the diagnosis of primary lung cancer (Qian et al., 2021).

Major limitations include the loss of epithelial markers, such as EpCam, on CTCs undergoing EMT, which may reduce the effectiveness of automated collection systems like CellSearch, leading to false-negative results (Andrikou et al., 2023; Gallo et al., 2017). Despite advances in detection methods, CTCs require highly sensitive detection due to their rarity compared to other types of blood cells (Pantel and Speicher, 2016). Additionally, CTCs may be lost during size-based filtration, especially when their size is similar to that of white blood cells (Andrikou et al., 2023). Variations in methods and thresholds used for detecting CTCs across different studies limit the comparability of results (Gallo et al., 2017).

### 7.3 Exosomes

Wang et al. (2018) aimed to identify exosomal protein markers specific to the plasma samples of metastatic NSCLC patients. Their investigation found that the lipopolysaccharide-binding proteins (LBPs) in exosomes can effectively distinguish between patients with metastatic and non-metastatic NSCLC, with a sensitivity of 83.1% and a specificity of 67% (Wang et al., 2018). Another group also focused their studies on plasma exosomes using the antibody-based Extracellular Vesicle Array (EV Array). They successfully detected surface-marker presenting exosomes, with a sensitivity of 0.75 and a specificity of 0.76, and they were able to classify the lung cancer patients with 75.3% accuracy (Jakobsen et al., 2015).

Exosomes are released from various tumor cells, which allows for a more accurate representation of tumor heterogeneity. They contain various cellular components that can be used to diagnose and monitor cancer progression, providing real-time information about patients’ cancer cells (Maqsood et al., 2024). Despite these advantages, there are technical limitations, associated with harvesting exosomes. Ultracentrifugation, a common method for isolating exosomes, is time-consuming, not cost-effective, and often results in a low yield (Gao et al., 2022). Additionally, filtration methods may lead to the loss of exosomes, as they can become trapped on filters (Gao et al., 2022). The similarity of exosomes to other extracellular vesicles (EVs) can also complicate their isolation, especially when the abundance of EVs in the sample is high (Cui et al., 2018).

### 7.4 microRNAs

Charkiewicz et al. (2023) conducted a microRNA profile using serum samples from 71 early-stage NSCLC patients and 47 non-cancerous pulmonary condition patients. Their studies identified 28 upregulated miRNAs in NSCLC compared to the control group. Subsequent analyses showed that these induced miRNAs are involved in NSCLC signaling pathways. Using the miRNA biomarkers, the authors were able to distinguish NSCLC from non-cancerous conditions with an accuracy of 0.837, a sensitivity of 0.806, and a specificity of 0.859 (Charkiewicz et al., 2023).

Another research team developed a reverse transcription PCR panel to test miRNAs from sputum samples, identifying miRs‐31‐5p and 210‐3p as biomarkers for NSCLC with a sensitivity of 65% and a specificity of 89% (Shen et al., 2014). The same group also tested three plasma miRNAs (miRs‐21‐5p, 210‐3p, and 486‐5p) for detecting NSCLC, achieving a sensitivity of 75% and a specificity of 85% (Shen et al., 2011). Additionally, they developed a panel containing both sputum and plasma miRNAs. With their training cohort of 76 NSCLC patients and 72 cancer‐free smokers, they reported sensitivities of 65.8%–75.0% and specificities of 83.3%–87.5% for the diagnosis of NSCLC (Liao et al., 2020). In their testing cohort of 56 NSCLC patients and 55 cancer‐free smokers, the authors demonstrated that their integrated panel of biomarkers, consisting of both sputum and plasma miRNAs, had higher sensitivity (85.5%) and specificity (91.7%) for the diagnosis of NSCLC compared to the individual panels alone (Liao et al., 2020).

As reviewed here, circulating miRNAs can serve as biomarkers for early diagnosis and prediction markers of cancer prognosis. One significant advantage of plasma miRNAs is their stability. They are stable in hot temperature and resistant to nuclease activity, indicating that they can be detectable in the bloodstream for extended periods (Mitchell et al., 2008). Additionally, miRNAs can be easily and cost-effectively detectable by real-time PCR, a relatively simple technique (Cui et al., 2019). Another key advantage is that miRNAs are highly conserved across species, allowing for easier transition from animal models to clinical studies (Ambros, 2004b).

Despite these advantages, the use of miRNAs as cancer biomarkers faces several major challenges, primarily due to technical limitations. miRNAs in the blood stream can interact with other cellular components, becoming fragile and making them difficult to isolate by centrifugation (Cheng et al., 2013). Another challenge is the normalization of data, which is complicated by the lack of reliable internal control miRNAs. For example, the presence of U6 in blood samples is often negative or marginal (Singh et al., 2016). Additionally, the levels of miR-16, commonly used as a control in many studies, can vary significantly depending on the cellular contexts (Wang and Chen, 2014). Furthermore, the precise mechanisms of miRNA secretion and regulation are not completely understood. This lack of understanding complicates the interpretation of miRNA levels and their role as biomarkers (Wang and Chen, 2014).

## 8 Conclusion

In conclusion, this review emphasizes the critical significance of circulating factors of NSLCL, highlighting the critical need for improving diagnostic and therapeutic strategies. The exploring of various circulating factors, including cancer-derived exosomes, ctRNA, CTCs, and ctDNA, highlights the potential of liquid biopsies as non-invasive alternatives for diagnosis and prognosis. Many circulating factors, including ctDNA, miRNA, and exosomes, can be readily detected from liquid biopsy samples using various diagnostic tools. Since the limitations of traditional tissue biopsies, which involve invasive procedures, are acknowledged, this review offers a comprehensive overview of various circulating biomarkers and approved diagnostic assays. Unlike tissue biopsies, liquid biopsies of blood and urine samples are more easily accessed. The exploration of various circulating biomarkers and their current research status, along with insights into approved diagnostic assays, provides a comprehensive overview of the evolving landscape in NSCLC diagnostics. Numerous clinical studies have provided compelling evidence for the successful application of liquid biopsy–based biomarkers and the utility of diagnostic tools for lung cancer.

Like many other diagnostic tools, detection sensitivity and false negatives might be considered limitations, especially when the concentration of biomarkers in liquid biopsy samples is low or subpopulations of CTCs are present. To establish clinically meaningful thresholds, more sensitive assays are being developed, including next-generation sequencing assays such as Guardant360 CDx (Bauml et al., 2022; Kwon et al., 2022) and LiquidHALLMARK (Choudhury et al., 2022b). Despite these current limitations, ongoing research into the complexities of circulating factors holds the promise of developing diagnostic markers and prognostic tools for lung cancer, offering expectation for improved outcomes and a more targeted approach to treatment. It is evident that these circulating factors, along with the diagnostic tools, hold strong potential for the development of personalized therapies for lung cancer.
